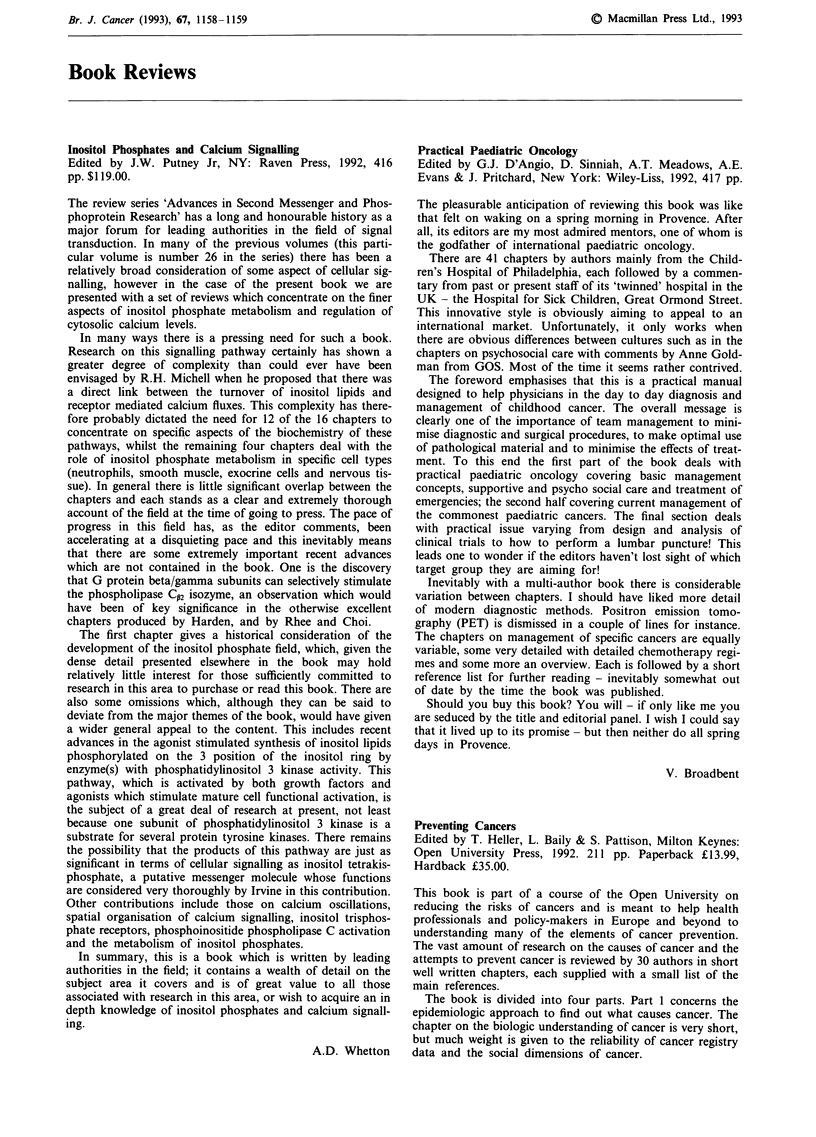# Practical Paediatric Oncology

**Published:** 1993-05

**Authors:** V. Broadbent


**Practical Paediatric Oncology**
Edited by G.J. D'Angio, D. Sinniah, A.T. Meadows, A.E. Evans & J. Pritchard, New York: Wiley-Liss, 1992, 417 pp.

The pleasurable anticipation of reviewing this book was like that felt on waking on a spring morning in Provence. After all, its editors are my most admired mentors, one of whom is the godfather of international paediatric oncology.

There are 41 chapters by authors mainly from the Children's Hospital of Philadelphia, each followed by a commentary from past or present staff of its 'twinned' hospital in the UK – the Hospital for Sick Children, Great Ormond Street. This innovative style is obviously aiming to appeal to an international market. Unfortunately, it only works when there are obvious differences between cultures such as in the chapters on psychosocial care with comments by Anne Goldman from GOS. Most of the time it seems rather contrived.

The foreword emphasises that this is a practical manual designed to help physicians in the day to day diagnosis and management of childhood cancer. The overall message is clearly one of the importance of team management to minimise diagnostic and surgical procedures, to make optimal use of pathological material and to minimise the effects of treatment. To this end the first part of the book deals with practical paediatric oncology covering basic management concepts, supportive and psycho social care and treatment of emergencies; the second half covering current management of the commonest paediatric cancers. The final section deals with practical issue varying from design and analysis of clinical trials to how to perform a lumbar puncture! This leads one to wonder if the editors haven't lost sight of which target group they are aiming for!

Inevitably with a multi-author book there is considerable variation between chapters. I should have liked more detail of modern diagnostic methods. Positron emission tomography (PET) is dismissed in a couple of lines for instance. The chapters on management of specific cancers are equally variable, some very detailed with detailed chemotherapy regimes and some more an overview. Each is followed by a short reference list for further reading – inevitably somewhat out of date by the time the book was published.

Should you buy this book? You will – if only like me you are seduced by the title and editorial panel. I wish I could say that it lived up to its promise – but then neither do all spring days in Provence.

V. Broadbent